# Contribution of Auger/conversion electrons to renal side effects after radionuclide therapy: preclinical comparison of ^161^Tb-folate and ^177^Lu-folate

**DOI:** 10.1186/s13550-016-0171-1

**Published:** 2016-02-09

**Authors:** Stephanie Haller, Giovanni Pellegrini, Christiaan Vermeulen, Nicholas P. van der Meulen, Ulli Köster, Peter Bernhardt, Roger Schibli, Cristina Müller

**Affiliations:** Center for Radiopharmaceutical Sciences ETH-PSI-USZ, Paul Scherrer Institut, 5232 Villigen-PSI, Switzerland; Laboratory for Animal Model Pathology, Institute of Veterinary Pathology, Vetsuisse Faculty, University of Zurich, 8057 Zurich, Switzerland; Laboratory of Radiochemistry, Paul Scherrer Institut, 5232 Villigen-PSI, Switzerland; Institut Laue-Langevin, 38042 Grenoble, France; Department of Radiation Physics, The Sahlgrenska Academy, University of Gothenburg, Sahlgrenska Universitetssjukhuset, 41345 Gothenburg, Sweden; Department of Chemistry and Applied Biosciences, ETH Zurich, 8093 Zurich, Switzerland

**Keywords:** Radionuclide therapy, ^161^Tb, ^177^Lu, Auger/conversion electrons, Radionephropathy, Kidney, Radiofolate

## Abstract

**Background:**

The radiolanthanide ^161^Tb has, in recent years, attracted increasing interest due to its favorable characteristics for medical application. ^161^Tb exhibits similar properties to the widely-used therapeutic radionuclide ^177^Lu. In contrast to ^177^Lu, ^161^Tb yields a significant number of short-ranging Auger/conversion electrons (≤50 keV) during its decay process. ^161^Tb has been shown to be more effective for tumor therapy than ^177^Lu if applied using the same activity. The purpose of this study was to investigate long-term damage to the kidneys after application of ^161^Tb-folate and compare it to the renal effects caused by ^177^Lu-folate.

**Methods:**

Renal side effects were investigated in nude mice after the application of different activities of ^161^Tb-folate (10, 20, and 30 MBq per mouse) over a period of 8 months. Renal function was monitored by the determination of ^99m^Tc-DMSA uptake in the kidneys and by measuring blood urea nitrogen and creatinine levels in the plasma. Histopathological analysis was performed by scoring of the tissue damage observed in HE-stained kidney sections from euthanized mice.

**Results:**

Due to the co-emitted Auger/conversion electrons, the mean absorbed renal dose of ^161^Tb-folate (3.0 Gy/MBq) was about 24 % higher than that of ^177^Lu-folate (2.3 Gy/MBq). After application of ^161^Tb-folate, kidney function was reduced in a dose- and time-dependent manner, as indicated by the decreased renal uptake of ^99m^Tc-DMSA and the increased levels of blood urea nitrogen and creatinine. Similar results were obtained when ^177^Lu-folate was applied at the same activity. Histopathological investigations confirmed comparable renal cortical damage after application of the same activities of ^161^Tb-folate and ^177^Lu-folate. This was characterized by collapsed tubules and enlarged glomeruli with fibrin deposition in moderately injured kidneys and glomerulosclerosis in severely damaged kidneys.

**Conclusions:**

Tb-folate induced dose-dependent radionephropathy over time, but did not result in more severe damage than ^177^Lu-folate when applied at the same activity. These data are an indication that Auger/conversion electrons do not exacerbate overall renal damage after application with ^161^Tb-folate as compared to ^177^Lu-folate, even though they result in an increased dose deposition in the renal tissue. Global toxicity affecting other tissues than kidneys remains to be investigated after ^161^Tb-based therapy, however.

## Background

The radiolanthanide terbium-161 (^161^Tb) has garnered increasing interest in recent years due to its favorable properties for medical application [1]. ^161^Tb has similar chemical properties to the clinically established lutetium-177 (^177^Lu), which enables stable coordination using a DOTA-chelator and, consequently, its application with well-established targeting agents [2]. The physical properties of ^161^Tb are also similar to those of ^177^Lu (T_1/2_ = 6.7 d, Eβ^−^_av_ = 134 keV, linear energy transfer (LET) of about 0.34 keV/μm [3]). ^161^Tb decays, with a half-life of 6.9 days, by the emission of low-energy β^−^-particles (Eβ^−^_av_ = 154 keV [3]) with a maximal tissue range of 0.29 mm and a LET of around 0.32 keV/μm, which is suitable for the treatment of metastasized malignancies. Importantly, ^161^Tb exhibits a co-emission of γ-radiation, enabling imaging using single photon emission computed tomography (SPECT) (Table 1). The decay process of ^161^Tb also releases a significant number of Auger/conversion electrons of an energy ≤50 keV (~12.4 e^−^, 46.5 keV per decay) [4], which are postulated to contribute to therapeutic anti-tumor effects [2]. This may be an advantage over ^177^Lu which emits a negligible number of Auger/conversion electrons in comparison (~1.11 e^−^, 1.0 keV per decay, Table 1) [4]. High-energy electrons emitted during β^−^-decay have a LET of about 0.2 keV/μm and a tissue range of 0.5–12 mm, suitable for larger metastases [5]. The high LET (~4–26 keV/μm) and short tissue range (~2–500 nm) of Auger/conversion electrons may be of particular value for the treatment of single tumor cells or tumor cell clusters [5]. This assumption has been corroborated by dosimetric calculations published by Uusijärvi et al., which clearly indicate the superiority of ^161^Tb over ^177^Lu for the treatment of small lesions (<200 μm) [6].

**Table 1:** Decay properties of ^161^Tb and ^177^Lu [3]

Radionuclide	^161^Tb	^177^Lu
Half-life	6.9 d	6.7 d
Eβ^−^_av_ (intensity)	154 keV (1.00)	134 keV (1.00)
Eγ (intensity)	25.7 keV (0.23)	112.9 keV (0.062)
	45 (0.18)*	208.4 keV (0.104)
	48.9 keV (0.17)	
	74.6 keV (0.10)	
E of Auger and conversion electrons (intensity)	0–0.1 keV (0.72)	0–0.1 keV (0.27)
	0.1–1 keV (7.38)	0.1–1 keV (0.55)
	1–10 keV (3.03)	1–10 keV (0.30)
	10–20 keV (0.42)	
	20–30 keV (0.18)	
	30–40 keV (0.39)	
	30–40 keV (0.72)	
	40–50 keV (0.23)	40–50 keV (0.05)
	0–50 keV (12.4)	0–50 keV (1.11)

*x-ray

Another advantage of ^161^Tb may be the fact that other Tb nuclides exist that emit diagnostic radiation only, potentially allowing pre-therapeutic dosimetry and monitoring therapy response with chemically identical imaging agents using SPECT (^155^Tb) and positron emission tomography (PET) (^152^Tb) [7].

The few studies in which ^161^Tb was applied thus far, focused on basic in vivo behavior of ^161^Tb-labeled biomolecules [8] and their effects on the malignant tissue in comparison to ^177^Lu [2, 9]. These preliminary therapy studies revealed that the therapeutic effect of ^161^Tb-labeled compounds was superior to the effect of their ^177^Lu-labeled counterparts, if applied at the same activities as indicated by dosimetric calculations [2].

Potential differences between undesired side effects of ^161^Tb and ^177^Lu have not been elucidated, however. The most critical organs in terms of side effects after radionuclide therapy are the bone marrow, populated by the highly radiosensitive hematopoietic cells, and the kidneys, which accumulate radioactivity of small molecular weight targeting agent at a high extent [10]. In the present study, we focused on the investigation of renal damage after exposure to therapeutic doses of ^161^Tb and ^177^Lu using a folate-based small molecular targeting agent.

The folate receptor (FR) has been found expressed in a wide variety of different tumor types, including cancer of the ovaries, endometrium, lungs, and kidneys [11]. It has therefore been exploited (pre)clinically for targeting with folate-based radioconjugates for nuclear imaging purposes [12, 13]. Following receptor binding on the cell surface, folic acid and its conjugates are internalized via FR-mediated endocytosis [14, 15]. This uptake mechanism makes folic acid also attractive for FR-targeted chemotherapy [16]. Recently, FR-targeted radionuclide therapy was exemplified using a DOTA-folate conjugate which was labeled with ^177^Lu [17]. It was shown that the application of ≥20 MBq of ^177^Lu-folate resulted in complete tumor remission in mice bearing KB tumor xenografts.

Targeted tumor therapy with folic acid-based radioconjugates is, however, known to present a risk of damage to the kidneys, since radiofolates accumulate specifically in the proximal tubule cells where the FR is expressed [11, 18]. This topic has been addressed recently, in a detailed investigation of radionephropathy after administration of ^177^Lu-folates with different in vivo distribution profiles [19].

The purpose of the current long-term study was to explore undesired side effects to the kidneys of mice after radionuclide therapy with high doses of ^161^Tb-folate and compare its nephrotoxic profile with the results obtained with ^177^Lu-folate. Histopathological changes of the renal tissue were examined after treatment with ^161^Tb-folate and compared to the results obtained with ^177^Lu-folate. Time-dependent monitoring of kidney function was performed over the whole period of investigation by the determination of blood plasma parameters, which are indicative for renal damage. Moreover, renal uptake of ^99m^Tc-DMSA was quantified using SPECT. This has proven to be a reliable method to determine kidney function in mice non-invasively after radionuclide therapy. The results of plasma parameters and SPECT were correlated with the data obtained from histological evaluation.

## Methods

### Preparation of ^161^Tb-folate and ^177^Lu-folate

The DOTA-folate conjugate (cm09), herein referred to as “folate,” was kindly provided by Merck & Cie (Schaffhausen, Switzerland) [17]. No-carrier-added ^161^Tb was produced at the spallation-induced neutron source (SINQ) at Paul Scherrer Institut (PSI) (Villigen-PSI, Switzerland) or at the high-flux reactor of the Institut Laue-Langevin (ILL, Grenoble, France) using various quantities of enriched ^160^Gd targets. Separation of ^161^Tb from the target material was carried out at PSI using cation exchange chromatography, as previously reported [1, 7]. No-carrier-added ^177^Lu was purchased from Isotope Technologies Garching (ITG GmbH, Garching, Germany). The radionuclidic purity of ^161^Tb and ^177^Lu, respectively, was ≥99 %. A HCl solution (0.05 M) of ^161^TbCl_3_ or ^177^LuCl_3_ (500 MBq, 100 μL) was mixed with sodium acetate (0.5 M, pH 8, 20 μL) to obtain a pH of ~4.5. After the addition of the folate conjugate (25 μL, 1 mM), the reaction mixture was incubated for 10 min at 95 °C. The resulting specific activity of the radiofolates was 20 MBq/nmol. Quality control was performed using HPLC as previously reported [17]. Before in vivo application, the radioconjugate solution was diluted with phosphate buffered saline (PBS) (pH 7.4) and Na-DTPA (10 μL, 5 mM, pH 5) was added for the complexation of potential traces of unreacted ^161^Tb(III) or ^177^Lu(III).

### Animal studies

The in vivo experiments were approved by the local veterinarian department and conducted in accordance with the Swiss law of animal protection. Female athymic nude mice (Crl:CD1-Foxn1^nu^, 5- to 7-week-old) were purchased from Charles River Laboratories (Sulzfeld, Germany). The mice received a folate-deficient rodent chow (ssniff Spezialdiäten GmbH, Soest, Germany), starting 1 week prior to the injection of the radiofolates. The folate-free diet was replaced by a standard rodent diet (Kliba Nafag, Kaiseraugst, Switzerland) 3 weeks after application of the radiofolates.

### Dosimetry

Dosimetric calculations were performed based on biodistribution data, which were previously obtained after the injection of ^161^Tb-folate and ^177^Lu-folate (2–3 MBq/0.5 nmol per mouse) using the same strain of mice (Crl:CD1-Foxn1^nu^) [2, 17, 19]. To estimate the mean absorbed radiation dose of ^161^Tb-folate and ^177^Lu-folate in the renal tissue, calculations were performed as follows: the cumulative radioactivity was calculated by fitting a bi-exponential curve to the non-decay-corrected biodistribution data (% IA/g). The area under the curve (MBq∙s) was determined by integrating the bi-exponential function to infinity. The mean absorbed dose to the kidneys was assessed for a kidney mass of 125 mg, having an elliptical shape and uniform activity distribution [20]. For the calculation of the absorbed fraction, the Monte Carlo code PENELOPE was used, resulting in an absorbed fraction of 0.93. In the simulation, detailed simulation mode was employed and the electron-absorption energy was 100 eV [21]. The mean absorbed dose (Gy/MBq) was calculated by multiplying the area under the curve normalized to 1 MBq injected activity (IA), with the absorbed fraction and the emitted energy per decay for ^161^Tb and ^177^Lu [3] and multiplied with a conversion factor.

### Long-term side effects after application of ^161^Tb-folate and ^177^Lu-folate

Effects of ^161^Tb-folate and ^177^Lu-folate, applied to non-tumor-bearing mice at therapeutic doses, were investigated over a period of 8 months. Control mice were intravenously (i.v.) injected with only saline (group A, *n* = 9). Three groups of six mice each were i.v. injected with ^161^Tb-folate of different activities (group B: 10 MBq, 0.5 nmol folate; group C: 20 MBq, 1 nmol folate; and group D: 30 MBq, 1.5 nmol folate). The same activites of ^177^Lu-folate were i.v. injected into three additional groups of six mice each (group E: 10 MBq, 0.5 nmol folate; group F: 20 MBq, 1 nmol folate; and group G: 30 MBq, 1.5 nmol folate). End-point criteria were defined as weight loss of >15 % of the initial body weight and/or signs of unease. Body weights were measured once a week for the determination of the relative body weight (RBW = *W*_*X*_/*W*_0_; with *W*_*X*_ = body weight at day *x* and *W*_0_ = body weight at day 0) and indicated as average RBW for each group.

### Determination of renal uptake of ^99m^Tc-DMSA using SPECT

The renal function of mice was monitored by the determination of the renal uptake of ^99m^Tc-DMSA using SPECT, as previously reported [22, 23]. DMSA (TechneScan®, Mallinckrodt, Petten, The Netherlands) was radiolabeled with ^99m^Tc (radionuclidic purity >99.9 %), obtained from a ^99^Mo/^99m^Tc-generator (Mallinckrodt, Petten, The Netherlands) at an activity concentration of 3 GBq in 5 mL. Quality control performed by TLC revealed a radiochemical purity of >95 %. SPECT acquisitions were performed 2 h after the injection of ^99m^Tc-DMSA (30-40 MBq per mouse) using a NanoSPECT/CT™ (Mediso Medical Imaging Systems, Budapest, Hungary) and Nucline Software (version 1.02, Bioscan Inc., Poway, USA). For this purpose, the energy window of ^99m^Tc was set to 140.5 ± 14 keV. The acquired data were reconstructed using HiSPECT software (version 1.4.3049, Scivis GmbH, Göttingen, Germany), and the renal uptake of ^99m^Tc-DMSA was determined, as previously reported, using the VivoQuant post-processing software (version 1.23, inviCRO Imaging Services and Software, Boston, USA) [19]. The determined activities were decay-corrected and expressed as percentage of injected activity (% IA) per kidney.

### Determination of blood plasma parameters

Blood urea nitrogen and creatinine were measured in weeks 8, 19, and 26 and at the day of euthanasia of the mice. Blood was taken from the sublingual vein of each mouse, collected in heparinized vials, and centrifuged. Blood plasma samples were analyzed using a Fuji Dri-Chem 4000i analyzer (Polymed Medical Center AG, Glattbrugg, Switzerland).

### Histological analysis

All animals were euthanized with CO_2_, followed by exsanguination. The kidneys were removed and fixed in 4 % buffered formalin (Formafix buffered 4 %, Switzerland AG, Hittnau, Switzerland). The tissue was trimmed, dehydrated, and embedded in paraffin wax. Sections of 3–5 μm thickness were prepared, mounted on glass slides, deparaffinized in xylene, and rehydrated through graded alcohols, before staining with hematoxylin and eosin (HE) for the histological examination. Renal injury was assessed by light microscopy. Glomerular, tubular, and interstitial lesions were evaluated separately using pre-defined criteria (Table 2), adapted from previously published studies [19, 24]. The values obtained for each compartment were summed to obtain a cumulative score. The cumulative score was then converted to a final score ranging from 0 to 5 (Table 3). No damage (score 0) indicated normal histology, while variably severe renal injury ranged from minimal damage (score 1), characterized by mildly reduced numbers of capillaries within the glomerular tuft and patchy tubular collapse, to severe renal damage (score 5), dominated by widespread glomerulosclerosis and prominent cortical parenchymal shrinkage. A phosphotungstic acid-hematoxylin (PTAH) staining was performed on tissues of selected mice for the identification of fibrin.

**Table 2:** Histological partial scoring, with each compartment (glomeruli, tubules, interstitium) evaluated separately

Partial score	Pathological changes
1	Glomeruli: mild deposition of PTAH-positive eosinophilic material (fibrin) and reduced number of capillaries in a few glomeruli.
	Tubules: <10 % of cortical tubules collapsed or degenerated.
	Interstitium: few small mononuclear cell infiltrates and/or minimal fibrosis in the cortex.
2	Glomeruli: mild/moderate fibrin deposition and reduced number of capillaries in numerous glomeruli.
	Tubules: 10–25 % of cortical tubules collapsed or degenerated.
	Interstitium: multifocal small mononuclear cell infiltrates and/or mild fibrosis in the cortex.
3	Glomeruli: marked fibrin deposition and reduced number of capillaries in numerous glomeruli.
	Tubules: 25–50 % of cortical tubules collapsed or degenerated.
	Interstitium: multifocal larger mononuclear cell infiltrates and/or moderate fibrosis in the cortex.
4	Glomeruli: marked fibrin deposition and reduced number of capillaries in numerous glomeruli; glomerulosclerosis in a few to several glomeruli.
	Tubules: 50–75 % of cortical tubules collapsed or degenerated; numerous dilated tubules.
	Interstitium: multifocal larger mononuclear cell infiltrates and/or moderate fibrosis in the cortex and medulla; mild/moderate parenchymal collapse.
5	Glomeruli: majority of glomeruli sclerotic.
	Tubules: >75 % of cortical tubules collapsed or degenerated; numerous dilated tubules.
	Interstitium: severe parenchymal collapse, affecting cortex and medulla.

**Table 3:** The partial scoring of each compartment (glomeruli, tubules, and interstitium) was summed to obtain the cumulative score

Cumulative score (glomeruli, tubules, interstitium)	Final score	Renal injury
0–0.9	0	No histological abnormality
1.0–2.9	1	Minimal
3.0–6.9	2	Mild
7.0–10.9	3	Moderate
11.0–13.9	4	Marked
14.0–15.0	5	Severe

The cumulative score was then converted to a final score indicating the degree of renal injury

### Statistical analysis

Data are presented as mean ± standard deviation. Statistics were conducted by using one-way ANOVA with Bonferroni’s multiple comparison post-test (GraphPad Prism, version 5.01).

## Results

### Radiolabeling

The radiolabeling of the folic acid conjugate was successfully achieved with ^161^Tb and ^177^Lu, respectively, at a specific activity of 20 MBq/nmol. Quality control performed by HPLC revealed a radiochemical yield >97 %.

### Dosimetric calculations

The mean absorbed kidney dose was estimated at 3.0 Gy/MBq for ^161^Tb-folate, of which ~76 % (2.3 Gy/MBq) was due to the β^−^-particles and ~24 % (0.7 Gy/MBq) due to Auger/conversion electrons. For ^177^Lu-folate, the total absorbed dose was estimated at 2.3 Gy/MBq, caused primarily by β^−^-particles, with only <1 % being due to Auger/conversion electrons (Table 4).

**Table 4:** Estimated mean absorbed kidney doses in mice after injection of ^161^Tb-folate and ^177^Lu-folate

	Control	^161^Tb-folate			^177^Lu-folate		
Group	A	B	C	D	E	F	G
Injected activity (MBq)	0	10	20	30	10	20	30
Mean absorbed kidney dose (Gy)	0	30	60	90	23	46	68

### Body weight and survival

**Fig. 1:**
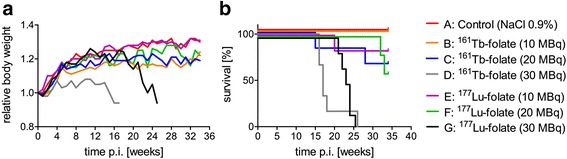
Average relative body weights (RBW) (**a**) and median survival (**b**) of mice from each group after the injection of different activities of ^161^Tb-folate and ^177^Lu-folate, respectively. RBWs are shown for each group for the time when at least two mice were alive (*n* ≥ 2)

All mice gained weight over the first month of the experiment, as a consequence of normal growth. Mice which were injected with 10 or 20 MBq of ^161^Tb-folate and ^177^Lu-folate, respectively, showed body weight gain within the first 6 weeks, followed by stabilization of the body weight in the range of 1.1 to 1.3 RBW (Fig. 1a). The majority of these mice (>50 %), as well as the control mice (groups A, B, C, E, and F), survived until the end of the study in week 34, and consequently, the survival time remained undefined (Fig. 1b). Four mice in these groups had to be euthanized in weeks 14, 19, 28, and 32, respectively, due to end-point criteria being reached, however. Mice that received high activities (30 MBq) of ^161^Tb-folate (group D) and ^177^Lu-folate (group G), respectively, lost body weight from week 15 on and showed signs of unease. This prompted euthanasia, in most of the cases, between weeks 15 and 26. The median survival time of ^161^Tb-folate treated mice (group D) was 17 weeks, whereas for ^177^Lu-folate treated mice (group G), the median survival was 23.5 weeks (Fig. 1b). This difference was determined not to be significant.

### Renal uptake of ^99m^Tc-DMSA

**Fig. 2:**
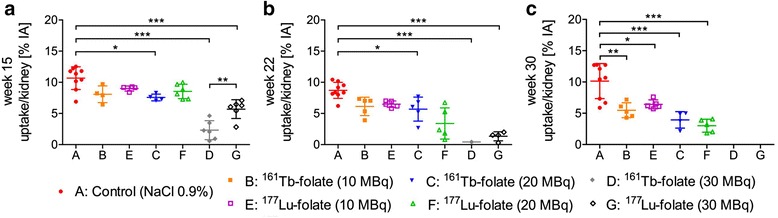
SPECT-based quantification of renal ^99m^Tc-DMSA uptake (% IA) in Week 15 (**a**), 22 (**b**), and 30 (**c**) after injection of ^161^Tb- and ^177^Lu-folate. *P < 0.05, **P < 0.01, ***P < 0.001

When the study reached week 3, all treated mice showed comparable renal uptake of ^99m^Tc-DMSA, which was in the same range as for the control mice of group A (data not shown). The mice injected with low activities of ^161^Tb-folate (group B; 10 MBq) and ^177^Lu-folate (group E; 10 MBq), respectively, showed reduced ^99m^Tc-DMSA uptake only 30 weeks after radiofolate injection, whereas no significant deviation from controls was observed at earlier time points (Fig. 2). In mice which were treated with 20 MBq of ^161^Tb-folate and ^177^Lu-folate, respectively, the uptake of ^99m^Tc-DMSA was 7.6 ± 0.5 % IA/kidney (group C) and 8.5 ± 1.2 % IA/kidney (group F) in week 15 and, thus, slightly lower than the value obtained for control mice (11 ± 1.8 % IA/kidney). In these mice (groups C and F), renal uptake of ^99m^Tc-DMSA decreased further in week 22 and week 30 (Fig. 2). The reduction of ^99m^Tc-DMSA was, however, comparable in mice injected with ^161^Tb-folate and ^177^Lu-folate (10 and 20 MBq), respectively.

Mice injected with the highest activity of ^161^Tb-folate (group D; 30 MBq) and ^177^Lu-folate (group G; 30 MBq) showed a significantly reduced renal uptake of ^99m^Tc-DMSA when compared to controls (group A) already in week 15 (Fig. 2). The determination of ^99m^Tc-DMSA uptake in week 15 revealed a significantly reduced value for mice injected with 30 MBq ^161^Tb-folate, compared to mice, which received 30 MBq ^177^Lu-folate. At later time points, the obtained values were, however, always comparable between these mice (groups D and G).

### Renal plasma parameters

Values of blood urea nitrogen and creatinine were not significantly different for control mice and mice treated with 10 MBq ^161^Tb-folate (groups B) over the entire period of investigation (Table 5). Mice which received 20 MBq ^161^Tb-folate (group C) showed slightly, but not significantly, increased levels of blood urea nitrogen as compared to control mice from week 26 on. Animals that received higher activities of ^161^Tb-folate (30 MBq, group D), however, showed significantly increased levels of blood urea nitrogen at terminal bleeding (42 ± 8.1 mM (*n* = 4) and > 50 mM (*n* = 2)) as compared to control mice (group A: 8.4 ± 1.4 mM). In the ^177^Lu-folate-treated groups, increasing levels of blood urea nitrogen were also observed in mice injected with 30 MBq (>50 mM). Moreover, significantly increased levels of blood urea nitrogen were also determined at terminal stage in mice that received only 20 MBq of ^177^Lu-folate (group G: 38 ± 4.1 mM; Table 5).

**Table 5:** Blood urea nitrogen and creatinine levels in blood plasma of mice injected with ^161^Tb-folate or ^177^Lu-folate

	Control	^161^Tb-folate			^177^Lu-folate		
Week	Group A	Group B: 10 MBq	Group C: 20 MBq	Group D: 30 MBq	Group E: 10 MBq	Group F: 20 MBq	Group G: 30 MBq
Blood urea nitrogen (mM)							
8	8.8 ± 0.8 (*n* = 7)	9.0 ± 1.3 (*n* = 5)	8.6 ± 0.6 (*n* = 6)	14 ± 7.5* (*n* = 6)	11 ± 0.8 (*n* = 6)	8.3 ± 0.6 (*n* = 6)	8.8 ± 0.6 (*n* = 4)
19	9.8 ± 0.6 (*n* = 9)	9.4 ± 0.6 (*n* = 6)	11 ± 1.6 (*n* = 5)	23 (*n* = 1)	10 ± 0.7 (*n* = 6)	9.3 ± 2.5 (*n* = 6)	26 ± 13*** (*n* = 6)
26	8.5 ± 0.8 (*n* = 9)	8.6 ± 1.6 (*n* = 6)	16 ± 12 (*n* = 5)	n.d.	9.2 ± 0.5 (*n* = 5)	21 ± 7.2 (*n* = 5)	>50*** (*n* = 1)
Terminal	8.4 ± 1.4 (*n* = 9)	9.2 ± 3.0 (*n* = 6)	>50 (*n* = 1)	>50 (*n* = 2)	10 ± 2.6 (*n* = 5)	38 ± 4.1*** (*n* = 4)	>50*** (*n* = 5)
			13 ± 3.2 (*n* = 5)	42 ± 8.1*** (*n* = 4)			
Creatinine (μM)							
8	<18 (*n* = 3)	<18 (*n* = 3)	<18 (*n* = 6)	<18 (*n* = 1)	<18 (*n* = 3)	<18 (*n* = 3)	<18 (*n* = 4)
	21 ± 3.5 (*n* = 4)	32 ± 1.4 (*n* = 2)		31 ± 14 (*n* = 5)	20 ± 1.0 (*n* = 3)	18 (*n* = 1)	
19	26 ± 9.6 (*n* = 8)	<18 (*n* = 2)	<18 (*n* = 3)	32 (*n* = 1)	<18 (*n* = 3)	<18 (*n* = 2)	25 ± 6.3 (*n* = 6)
		25 ± 7.3 (*n* = 4)	21 ± 4.9 (*n* = 2)		22 ± 0.6 (*n* = 3)	18 ± 0 (*n* = 3)	
26	<18 (*n* = 4)	<18 (*n* = 4)	<18 (*n* = 2)	n.d.	30 ± 19 (*n* = 5)	33 ± 7.6 (*n* = 5)	46 (*n* = 1)
	20 ± 1.7 (*n* = 4)	22 ± 0.7 (*n* = 2)	33 ± 5.9 (*n* = 3)				
Terminal	<18 (*n* = 3)	21 ± 3.8 (*n* = 6)	<18 (*n* = 1)	77 ± 17* (*n* = 6)	25 ± 7.6 (*n* = 5)	<18 (*n* = 2)	107 ± 35** (*n* = 5)
	24 ± 5.1 (*n* = 6)		51 ± 48 (*n* = 5)			63 ± 46 (*n* = 2)	

The blood plasma parameters were determined in Week 8, 19, 26 and before euthanasia (terminal)

Abbreviations: *n.d.* not determined (already euthanized), *n* number of mice

Statistics: comparison of treated animals to control; *P < 0.05, **P < 0.01, ***P < 0.001

Detection limits: blood urea nitrogen: 50 mM; creatinine: 18 μM

Application of high activities of ^161^Tb-folate (group D, 30 MBq) resulted in a significant increase of creatinine plasma levels (77 ± 17 μM), as compared to control mice of group A (24 ± 5.1 μM (*n* = 6), <18 μM (*n* = 3)) at terminal state and the same observation (creatinine level of 107 ± 35 μM) was also made for ^177^Lu-folate treated mice (group G, 30 MBq). In mice which received 20 MBq ^161^Tb-folate (group C) and ^177^Lu-folate (group F), respectively, slightly, but not significantly, increased levels of creatinine were determined in Week 26 and at terminal bleeding.

### Renal histopathology

**Fig. 3:**
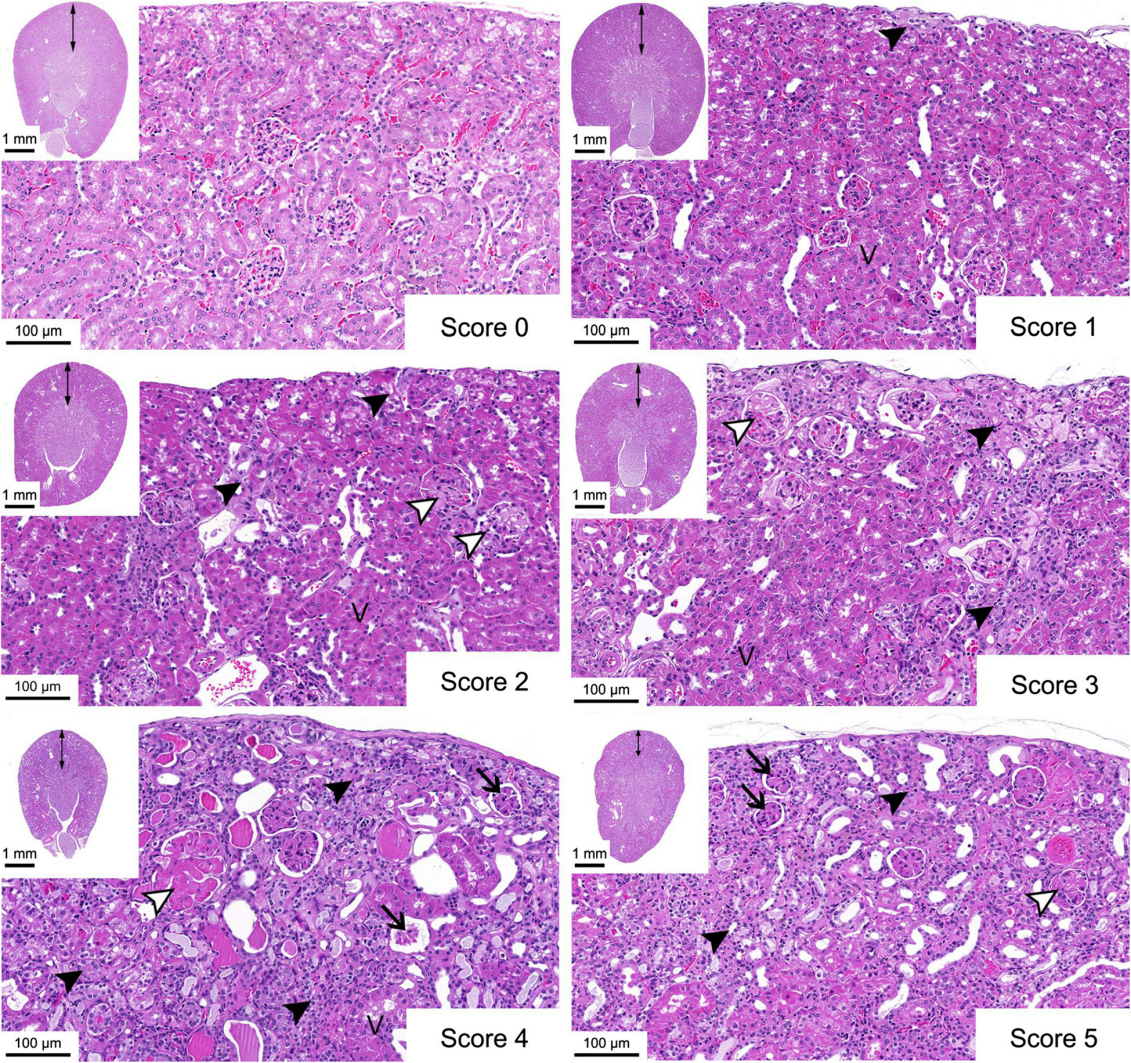
Histology (HE staining) of radiation-induced nephropathy in mice. Representative histological images (×20 magnification) of kidneys from untreated controls (final score 0) and progressively severe kidney damage (final scores 1–5) from mice injected with different activities of ^161^Tb-folate (▲ = localized tubular collapse; v = viable tubules; Δ = enlarged glomeruli with reduced numbers of capillaries; → = glomerulosclerosis). In the *insets*, cross sections of the kidneys are presented (×2 magnification) indicating gradual thinning of the cortex (↔) with increasing scores

The application of ^161^Tb-folate and ^177^Lu-folate resulted in a dose-dependent renal damage consistent with radiation nephropathy (Table 6). The kidneys of the control animals did not show histological abnormality and received a final score of 0. The renal tissue of mice injected with increasing activities of radiofolates showed progressively more severe kidney damage, which was graded with final scores between 1 and 5 (Fig. 3). For the mice treated with ^161^Tb-folate, final scores of 1 (group B, 10 MBq), 3 (group C, 20 MBq), and 4 (group D, 30 MBq) were determined. In the case of ^177^Lu-folate application, renal damage was scored with 2 (group E, 10 MBq), 3 (group F, 20 MBq), and 4 (group G, 30 MBq). Lower scores were characterized by localized tubular collapse, high proportions of viable tubules, and enlarged glomeruli with reduced numbers of capillaries. These changes became more prominent in the severely injured kidneys, where occasional to frequent glomerulosclerosis was also observed (Fig. 3). Moreover, a gradual thinning of the cortex with increasing amounts of applied radiofolate was observed during the investigation of renal cross sections (Fig. 3).

**Table 6:** Histological evaluation of renal radiation injury in mice injected with ^161^Tb-folate or ^177^Lu-folate, indicated with partial scores for glomeruli, tubules, and interstitium

	Control	^161^Tb-folate			^177^Lu-folate		
	Group A^a^(*n* = 9)	Group B: 10 MBq	Group C: 20 MBq	Group D: 30 MBq	Group E: 10 MBq	Group F: 20 MBq	Group G:30 MBq
		(*n* = 5)^a^	(*n* = 5)^a^	(*n* = 2)^b^	(*n* = 5)^a^	(*n* = 4)^a^	(*n* = 5)^b^
Glomeruli	0	1.1	3.4	5.0	2.0	3.8	5.0
Tubules	0	0.9	2.4	4.5	1.5	3.3	4.2
Interstitium	0	0.4	1.8	4.0	0.4	3.3	4.6
Cumulative score	0	2.4	7.6	13.5	3.9	10.4	13.8
Final score	0	1	3	4	2	3	4

The sum of the partial scores determined the cumulative scores, which were then converted to the final score as a measure for the different degrees of renal injury

^a^Mice euthanized in Week 34

^b^Mice euthanized between Weeks 15 and 26

### Correlation of kidney function tests and renal histopathology

**Fig. 4:**
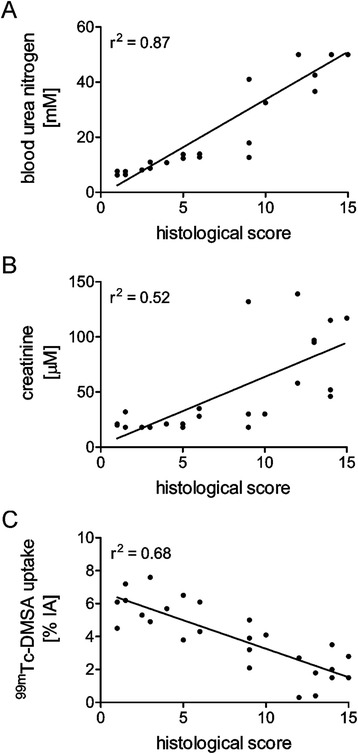
Correlation of individual results for kidney function tests and histological scores of the corresponding renal tissue sample. **a** Correlation of the histological scores and terminal blood urea nitrogen levels; **b** Correlation of the histological scores and terminal creatinine levels; **c** Inverse correlation of the histological scores and ^99m^Tc-DMSA uptake in the kidneys

Retrospectively, renal function parameters determined shortly before euthanasia were correlated with the histology scores on an individual basis (Fig. 4). Increasing blood urea nitrogen levels correlated (*r*^2^ = 0.87) well with rising scores of radiation nephropathy (Fig. 4a). The creatinine levels indicated, however, only a slight trend in the same direction as they showed poor correlation with the scoring results (*r*^2^ = 0.52). A relatively good correlation (*r*^2^ = 0.68) was found between the determination of renal damage using ^99m^Tc-DMSA/SPECT and the overall score determined by histopathological evaluation of the renal damage.

## Discussion

A cumulative renal dose of >25 Gy as a result of ^177^Lu- or ^90^Y-based radionuclide therapy comprises a high risk of renal damage in patients [25]. In contrast, radionuclide therapy based on Auger/conversion electrons using ^111^In-octreotide did not impair kidney function up to a renal dose of 45 Gy [26]. It was hypothesized that, because of their short tissue range, Auger/conversion electrons are unable to damage the radiosensitive glomeruli and potential cell damage would, therefore, be limited to the more radioresistant tubular cells, in which the radioconjugates accumulate [26]. Thus, it was speculated that the accumulation of activity into specific organ structures, yielding a local energy deposit for short-ranged electrons, might be the reason for the reduced impairment of renal function after therapy with short-ranged Auger/conversion electrons, as compared to treatment with the long-ranged β^−^-particles.

In this work, we investigated potential radionephrotoxicity caused by therapeutic doses of ^161^Tb-folate in mice. The data were compared with those previously obtained with ^177^Lu-folate [19]. The slightly higher β^−^-energy of ^161^Tb (Eβ^−^_av_ = 154 keV) compared to ^177^Lu (Eβ^−^_av_ = 134 keV) and, predominantly, the co-emitted Auger/conversion electrons of ^161^Tb resulted in about 1.3-fold increased mean absorbed kidney dose (3.0 vs. 2.3 Gy/MBq), if the same activities of ^161^Tb-folate and ^177^Lu-folate were employed. The Auger/conversion electrons contributed with about 24 % of the mean absorbed renal dose upon the injection of ^161^Tb-folate. In this experimental setting, the applied mean absorbed renal dose of ^161^Tb-folate ranged from 30 Gy (10 MBq) to 90 Gy (30 MBq). The corresponding dose for ^177^Lu-folate, applied at the same activity, ranged from 23 Gy (10 MBq), as the safe limit, to 68 Gy (30 MBq), which is known to induce renal damage [19]. The absorbed doses that are presented refer to the whole mean absorbed kidney dose, as it is most often presented in clinical studies [10]. Furthermore, an even more detailed description of the activity deposited into the proximal tubular cells might differentiate the absorbed dose profiles for ^161^Tb and ^177^Lu due to different emission of low-energy electrons. A detailed nephron-based model of the absorbed dose to the kidneys has recently been described by Hobbs et al. for α-emitters [27]. A similar approach will be utilized in the future for the characterization of the absorbed dose profiles for ^161^Tb and ^177^Lu, together with detailed autoradiography studies at different time points after injection.

The injection of high activities of ^161^Tb-folate (30 MBq), which resulted in a mean absorbed kidney dose of 90 Gy (and as discussed above, might be 1.5-fold higher, if all activity is considered to be located in the cortex), was not well tolerated and resulted in body weight loss, which required euthanasia in the second half of the study (Fig. 1). End-point criteria were reached earlier in ^161^Tb-folate treated mice (group D) than in mice which received the same activities of ^177^Lu-folate (30 MBq, ~68 Gy, group G). The severity of renal damage, assessed by kidney function tests and histological evaluation, was, however, comparable between mice injected with ^161^Tb-folate (group D) and ^177^Lu-folate (group G). The reason why the relative body weight and survival rate did not correlate well with the severity of renal damage in these high-dose treatment groups remains unknown and might involve other factors referring to global toxicity, which were not investigated in this study.

^99m^Tc-DMSA uptake studies performed 3 weeks after radiofolate application showed comparable results in all groups indicating normal kidney function. In Week 15, impaired kidney function was observed in mice which were exposed to 20 and 30 MBq of ^161^Tb-folate or 30 MBq of ^177^Lu-folate (groups C, D, and G) based on the results obtained with ^99m^Tc-DMSA (Fig. 2a). About 7 months after radiofolate treatment, the mice which were exposed to lower renal doses (groups B, E, and F) also showed a significantly reduced uptake of ^99m^Tc-DMSA in comparison to untreated control mice (Fig. 2c). Parallel analyses of blood urea nitrogen and creatinine plasma levels confirmed the dose-dependent impairment of kidney function which was observed in studies with ^99m^Tc-DMSA-based SPECT.

The same activity of ^161^Tb-folate and ^177^Lu-folate did not cause significant differences regarding renal uptake of ^99m^Tc-DMSA and rising levels of plasma parameters, indicative for kidney function, over the whole period of investigation. This observation indicates that the impairment of renal function runs in parallel for ^161^Tb-folate and ^177^Lu-folate. A significantly lower renal accumulation of ^99m^Tc-DMSA was only found in week 15 for mice treated with 30 MBq ^161^Tb-folate (Group D) compared to mice injected with 30 MBq ^177^Lu-folate (Group G). Other than that, the determination of blood urea nitrogen and creatinine plasma levels did not confirm a significant difference of the renal function in mice of groups D and G at any time of the study. In contrast, at terminal state, there was even a tendency of increased blood urea nitrogen and creatinine levels in ^177^Lu-folate-treated mice (Group G) as compared to the levels determined in ^161^Tb-folate-treated mice (Group D).

Histopathological changes were investigated with renal tissue of euthanized mice, when an end-point criterion was reached or after termination of the study in Week 34. A reduced renal mass and thinning of the renal cortex was observed along with increasing tissue damage in the renal cortex, where radiofolates accumulate because of FR-expression in the proximal tubular cells [11, 18]. Evaluation of the partial and cumulative scores in the tubules, glomeruli, and interstitium showed equal distribution and location of renal damage independent on the radionuclide which was employed (Table 6). This means that the application of ^161^Tb-folate resulted in renal damage that was comparable to that observed after the application of ^177^Lu-folate at the same activity, in spite of the 1.3-fold higher dose which was deposited in the case of ^161^Tb-folate. These data are in line with the hypothesis that the additional dose of ^161^Tb-folate, due to Auger/conversion electrons, has no adverse effect on renal function and morphology. This theory appears plausible for radiopharmaceuticals that accumulate primarily in tubular cells, as it is the case for folate- and peptide-based radioconjugates [18, 28–30]. Auger/conversion electrons deposit energy over subcellular dimensions only and, hence, no other parts of the kidneys will be reached other than the tubules. Our observation is in agreement with the theory that in the case of kidneys which is defined as flexible tissue (in contrast to hierarchical tissues [31]), the irradiated volume and affected suborgan unit are critical factors for the final renal injury [32, 33].

The above reported results are corroborated by the fact that values determined for blood urea nitrogen levels correlated well with the histopathological analysis which was performed in an independent setting (Fig. 4). The value of creatinine levels for predicting renal damage appears to be questionable due to high variability and poor correlation with the histopathological changes. The unreliable value of creatinine as an indicator for renal damage was previously reported in the literature [34]. From a methodological point of view, ^99m^Tc-DMSA/SPECT is undoubtedly the most sensitive method for non-invasive monitoring of renal function.

## Conclusions

The functional and histopathological analysis of the kidneys after application of ^161^Tb-folate revealed a dose-dependent damage which was comparable to the damage caused by ^177^Lu-folate applied using the same activity. These observations are in line with the hypothesis that Auger electrons and low energy conversion electrons do not result in additional renal injury, as previously observed during therapeutic application of ^111^In-octreotide in patients. The confirmation of these results, in more detailed future studies, will support the clinical application of ^161^Tb-based therapy, which could contribute to increase the success rate of the treatment without causing additional renal side effects.

## Compliance with ethical standards

This article does not contain any studies with human participants performed by any of the authors.

All applicable international, national, and/or institutional guidelines for the care and use of test animals were followed.

## Abbreviations

DMSA: dimercaptosuccinic acid
DTPA: diethylene triamine pentaacetic acid
FR: folate receptor
HE: hematoxylin and eosin
HPLC: high performance liquid chromatography
IA: injected activity
PBS: phosphate buffered saline pH 7.4
PET: positron emission tomography
RBW: relative body weight
SPECT: single photon emission computed tomography
TLC: thin layer chromatography
